# Multiple Domains of Human CLASP Contribute to Microtubule Dynamics and Organization In Vitro and in *Xenopus* Egg Extracts

**DOI:** 10.1002/cm.21005

**Published:** 2012-01-25

**Authors:** Kieren Patel, Eva Nogales, Rebecca Heald

**Affiliations:** 1 Department of Molecular and Cell Biology, University of CaliforniaBerkeley, California; 2 Life Science Division, Lawrence Berkeley National LaboratoryBerkeley, California; 3 Howard Hughes Medical Institute, University of CaliforniaBerkeley, California

**Keywords:** microtubule plus end-binding protein, cytoplasmic linker associated proteins, microtubule dynamics, mitotic spindle assembly, *Xenopus* egg extract

## Abstract

Cytoplasmic linker associated proteins (CLASPs) comprise a class of microtubule (MT) plus end-binding proteins (+TIPs) that contribute to the dynamics and organization of MTs during many cellular processes, among them mitosis. Human CLASP proteins contain multiple MT-binding domains, including tumor over-expressed gene (TOG) domains, and a Ser-x-Ile-Pro (SxIP) motif known to target some +TIPs though interaction with end-binding protein 1 (EB1). However, how individual domains contribute to CLASP function is poorly understood. We generated full-length recombinant human CLASP1 and a series of truncation mutants and found that two N-terminal TOG domains make the strongest contribution to MT polymerization and bundling, but also identified a third TOG domain that further contributes to CLASP activity. Plus end tracking by CLASP requires the SxIP motif and interaction with EB1. The C-terminal coiled-coil domain mediates dimerization and association with many other factors, including the kinetochore motor centromere protein E (CENP-E), and the chromokinesin Xkid. Only the full-length protein was able to rescue spindle assembly in *Xenopus* egg extracts depleted of endogenous CLASP. Deletion of the C-terminal domain caused aberrant MT polymerization and dramatic spindle phenotypes, even with small amounts of added protein, indicating that proper localization of CLASP activity is essential to control MT polymerization during mitosis. © 2012 Wiley Periodicals, Inc

## Introduction

Microtubule (MT) assembly and organization are highly regulated by many associated proteins, including a subset that selectively localize to growing plus ends (+TIPs) [Galjart,^2010^]. Cytoplasmic linker associated proteins (CLASPs) make up a highly conserved class of +TIPs thought to promote polymerization of specific subsets of MTs and link them to cellular structures [Jiang and Akhmanova,^2011^]. CLASPs have a broad range of functions in cell organization, motility and mitosis, localizing to the Golgi, cell cortex, mitotic spindle midzone, and kinetochores [Mimori-Kiyosue et al.,^2006^; Sousa et al.,^2007^; Liu et al.,^2009^; Miller et al.,^2009^]. Recent studies of the *Schizosaccharomyces pombe* homolog Cls1p/Peg1 showed that CLASP can promote MT rescues and suppress catastrophes independent of other proteins [Al-Bassam et al.,^2010^], but molecular mechanisms behind CLASP function and targeting to MT plus ends and cellular structures remain unclear.

Two distinct modes of autonomous MT plus end tracking in vitro are represented by the end-binding protein 1 (EB1) family and the *Xenopus* MT associated protein of 215 kD (XMAP215) family. Whereas, EB1 contains an N-terminal calponin homology domain that recognizes MT plus end features and a C-terminal dimerization domain, XMAP215 binds the MT lattice and utilizes tandem tumor over-expressed gene (TOG) domains to deliver tubulin dimers to growing plus ends [Al-Bassam et al.,^2007^; Brouhard et al.,^2008^; Vitre et al.,^2008^]. Numerous other +TIPs target to MT plus ends by interacting with EB1, either through cytoskeletal associated protein glycine-rich (CAP-Gly) domains, such as the cytoplasmic linker protein 170 (CLIP-170) and p150-GLUED or through Ser-x-Ile-Pro (SxIP) motifs, including adenomatous polyposis coli, microtubule actin crosslinking factor 2 (MACF2), and mitotic centromere-associated kinesin (MCAK) [Plevin et al.,^2008^; Honnappa et al.,^2009^]. Interestingly, CLASP contains TOG and TOG-like domains in addition to SxIP motifs [Galjart,^2005^; Slep,^2009^,^2010^]. Although the *S. pombe* CLASP associates with CLIP-170 and EB1, the homodimer is sufficient to recruit tubulin to growing MTs through its N-terminal TOG domains in the absence of other proteins [Grallert et al.,^2006^; Al-Bassam et al.,^2010^].

CLASP plays important roles during mitosis and is directed to the spindle midzone and kinetochores through association with the bundling protein regulator of cytokinesis 1 (PRC1) and the centromere associated protein E (CENP-E), respectively [Hannak and Heald,^2006^; Liu et al.,^2009^]. Mutant analysis and RNA interference (RNAi) of the *Drosophila* version, multiple asters (MAST)/Orbit, revealed that it is required for chromosome alignment, kinetochore-MT attachment, and maintenance of spindle bipolarity [Mathe et al.,^2003^]. Photobleaching and microsurgery revealed that CLASP is involved in polymerization at plus ends essential for MT poleward flux [Maiato et al.,^2005^]. Further evidence supporting a role for CLASP in mitosis comes from studies in human cells and *Caenorhabditis elegans* embryos [Maiato et al.,^2003^; Cheeseman et al., 2005], but how different domains of CLASP contribute to its function is poorly understood. We previously found *Xenopus* CLASP, also called Xorbit, to be essential for MT stabilization, both at kinetochores and along chromosome arms or chromatin in egg extracts [Hannak and Heald,^2006^]. Spindles formed in CLASP-depleted extracts were small and misshapen, and MTs disappeared during anaphase, indicating that *Xenopus* CLASP plays a key role in maintaining MT polymerization and connection to chromosomes as they align and segregate.

Mammalian CLASP exists in two isoforms, CLASP1 and 2, which exhibit the same localization in mitosis and appear to play redundant roles [Mimori-Kiyosue et al.,^2006^]. Transfection of truncated versions of CLASP1 and 2 have been used to identify regions required for MT association along the lattice and plus end, and binding to mitotic structures including the kinetochore, centrosomes, and midbody [Maiato et al.,^2003^; Mimori-Kiyosu et al.,^2005^]. In vitro studies with purified CLASP has thus far been limited to the fission yeast protein Cls1p/Peg1 [Al-Bassam et al.,^2010^]. In this paper, we use a series of purified CLASP1 proteins to provide biochemical insight into the structure and activity of human CLASP, its effects on MT dynamics, MT plus end targeting, interaction with other cellular factors, and its function in the context of the meiotic spindle in *Xenopus* egg extracts.

## Experimental Procedures

### Cloning and Protein Expression of CLASP1 and Fragments

Full-length CLASP1 and smaller constructs, designed based on structure prediction of domain boundaries (Supporting Information Fig. S1), were PCR amplified using PFX Taq polymerase (Invitrogen, Grand Island, NY) from a cDNA template provided by I.M.A.G.E. consortium (Clone AAI32724.1). Amplicons, flanked by ligation independent cloning (LIC) sites, were cloned into a PIEX/BAC-1 (EMD) containing a StrepII tag for N-terminal fusion. StrepII-CLASP1 and fragments were subsequently cloned into a modified pFAST-bac vector (Invitrogen), containing additional LIC sites and an eGFP-10X histidine sequence for C-terminal fusion. Cloning products were analyzed after transformation into DH5α cells (Invitrogen) and confirmed by plasmid sequencing. Protein expression in SF9 insect cells (Expression Systems, Woodland, CA) was performed as described by the manufacturer's protocol (Invitrogen). Protein was expressed in 1 L of cells (1 × 10^6^ cells/mL) for 48 h at 28°C and harvested for purification.

CLASP proteins were affinity purified making use of both the StrepII and polyhistidine tags. Insect cells were harvested, washed once in cold PBS (phosphate buffered saline solution, pH 7.4), pelleted (1000 RPM, JS-4.0 rotor; Beckman, Brea, CA), and flash frozen in liquid nitrogen. The cell pellet was lysed using a glass homogenizer and lysis buffer (100 mM HEPES (4-(2-hydroxyethyl)-1-piperazineethanesulfonic acid)), pH 7.5, 500 mM NaCl, 5% glycerol, 0.2% Tween-20, 1 mM dithiothreitol (DTT), ethylenediaminetetraacetic acid (EDTA), and 1 tablet of protease inhibitor cocktail (Roche, Indianapolis, IN) per 50 mL volume of lysate). Crude lysate was clarified at 35,000 × *g* for 20 min. C-terminal histidine tagged proteins were isolated by metal affinity chromatography (NTA-agarose His-Select affinity gel, GE, Piscataway, NJ) using the manufacturer's protocol. Protein was eluted using 400 mM imidazole. StrepII affinity purification was performed by applying Streptactin affinity gel (Qiagen, Valencia, CA) to pooled fractions of eluted protein from the nickel gel elutions. After 1 h incubation at 4°C, the gel was washed with cold 1× PBS and eluted with elution buffer (100 mM HEPES, pH 7.5, 150 NaCl, 0.2% Tween-20, 1 mM DTT, EDTA, and 10 mM d-biotin; Sigma, St. Louis, MO). Fractions were analyzed by sodium dodecyl sulfate polyacrylamide gel electrophoresis (SDS-PAGE), stained with Sypro-Ruby, imaged using an α-Imager (Bio-rad, Philadelphia, PA) and flash frozen, and stored in liquid nitrogen for subsequent assays.

### Negative Stain Electron Microscopy

Full-length human CLASP was visualized using electron microscopy (EM) of negatively stained samples. Purified protein (3.2 mg/mL) was diluted in elution buffer (50 mM HEPES, pH 7.5, 150 mM NaCl, 1 mM DTT, 0.1% Tween-20, and 10 mM d-biotin) to a concentration of ∼9 μg/mL. A 400-mesh copper grid (Ted Pella, Redding, CA) overlaid with a very thin continuous carbon layer was gently glow discharged and 5 μL of the diluted protein was applied to the grid. After a 5 min adsorption, the protein was blotted away from the grid with filter paper (Whatman No.1) leaving a thin layer of solution on the grid. The grid was then picked-up and gently floated on a 5 μL drop of 4% uranyl-acetate solution on a parafilm support. After 30 s, the grid was picked up and the stain blotted from the grid and the sample was floated again on 5 μL drop of fresh stain for 2 min. After blotting, the grid was left to dry for 2 min. The negative stain sample of CLASP was imaged using a Tecnai12 transmission electron microscope (TEM) and images recorded on a Gatan CCD camera at ∼−1.5 μm defocus.

Tubulin (Cytoskeleton, Denver, CO) in MT polymerization buffer was incubated at 37°C for 1 h in the presence of 1 mM guanosine triphosphate (GTP), 1 mM DTT, and 200 μM Taxol. The MT solution was then diluted 1:100 into the same buffer and deposited on an EM grid. After 2 min, the MT solution was blotted off the grid, CLASP diluted from stock to 50 μg/mL was then pipetted on and allowed to adsorb for 2 min. This last step was repeated twice. The grid was then stained with uranyl acetate and imaged as described above.

### Gel Filtration of CLASP

Protein samples were diluted in low salt solution similar to elution buffer (50 mM HEPES, pH 7.5, 50 mM NaCl, 1 mM DTT, 0.1% Tween-20, and 10 mM d-biotin). Since the protein begins to aggregate at lower salt concentrations, the samples were ultracentrifuged for 20 min at 90K rpm (TLA100.1 rotor; Beckman) to clarify the sample before loading on the column. A Superdex 200 HR/30 was equilibrated with the dilution buffer and 100 μL of each sample was analyzed. Traces were compared to standards also run on the column to allow estimation of molecular weights.

### CLASP-MT Pelleting Assays

To examine the binding affinity of individual CLASP fragments, concentrations of taxol stabilized MTs ranging from 0 to 4 μM were incubated with a fixed concentration of CLASP (0.5 μM) in a buffer consisting of BRB80 (80 mM PIPES (1,4-piperazinediethanesulfonic acid); 1 mM EGTA; and 1 mM MgCl_2_, pH 6.8 Stabilization Buffer (100 mM Glycerophostphate, pH 7.0; 2 mM), 80 mM NaCl, 1 mM DTT, and 1 mM GTP. Samples were incubated for 15 min and then sedimented by centrifugation at 90K rpm. After the supernatant was removed, the pellet was resuspended on ice for 30 min with sample buffer. The pellet and supernatant fractions were analyzed by SDS-PAGE, stained with Sypro Ruby stain (Bio-rad), and imaged with an α Imager (Bio-rad). Band intensities were quantified using ImageJ (NIH) and plotted to construct an affinity curve from which the kd was calculated.

To further investigate the nature of interaction, sensitivity of CLASP binding was tested with both salt and subtilisin treated MTs. Taxol MTs were prepared as described above. For salt sensitivity tests, 5.0 μM CLASP was incubated with 1 μM MTs and sedimented. The pellet was resuspended and incubated with 25–500 mM NaCl in BRB80 buffer for 20 min. MTs were sedimented and analyzed as previously described.

Taxol-stabilized MTs (2 mg/mL) were treated with subtilisin (80 μg/mL) at 37°C for 10 min. MTs missing the C-terminal tails (or E-hooks) of both α- and β-tubulin were produced by 120 min of digestion under similar conditions. In both cases, subtilisin cleavage was halted by the addition of 4 mM pimindimethyl sulfoxide for 20 min at 25°C, and the MTs were then pelleted at 100,000 × *g* for 15 min. The pellet was washed once and resuspended to an approximate concentration of 1 μM tubulin in BRB80 containing 1 mM GTP and 20 μM taxol. CLASP (5 μM) was added and incubated for 15 min, after which the sample was sedimented and analyzed as previously described.

For the cold pelleting assay, tubulin was first polymerized at 37°C in the presence of CLASP1–662 protein in a similar reaction to that described for the electron microscopy analysis of full-length CLASP bound to MTs (see above). After polymerization for 60 min, the reaction was placed in ice for 30 min. The samples were then sedimented at 90K rpm for 20 min (rotor TLA100.1; Beckman). The supernatant was removed and 4× SDS sample buffer added. Sample buffer was also added to the pellet, vortexed for 2 min, and incubated on ice for 30 min before analysis by SDS-PAGE.

### Light Scattering Assay for Measurement of MT Polymerization and Bundling Kinetics

In these assays, a fluorimeter (Hitachi XW-100) was used with excitation and emission wavelengths set at 350 nm and a slit width of 1.5 nm. After clarification by centrifugation at 90K for 20 min to remove protein aggregates, CLASP protein (50 μg/mL) in MT polymerization buffer (80 mM PIPES, 1 mM MgCl_2_, and 0.5 mM EGTA) supplemented with 1 mM GTP, 1 mM DTT, and 80 mM NaCl was added to a glass cuvette. 15 μM bovine tubulin (Cytoskeleton) was then added, and the cuvette placed in a heated chamber (∼32°C) within the fluorimeter. Data was collected for 25 min, with absorbance measured every 10 s.

### Depletion/Rescue and CLASP Addition to *Xenopus* Egg Extract

*Xenopus* laevis egg extracts arrested in metaphase of meiosis II by cytostatic factor (CSF) were prepared as described [Hannak and Heald,^2006^]. Immunodepletion of Xorbit was accomplished using 10 mg of α-Xorbit antibody coupled to 50 μL protein A-Dynabeads (Dynal), which were incubated in 150 μL of CSF extract. Nonspecific rabbit IgG antibody (Sigma-Aldrich) was used as a mock-depletion control. Two rounds of depletion were performed. The level of depletion was tested by Western blot with 1 μg/mL of α-Xorbit antibody. Full-length CLASP, CLASP662–1463 or CLASP662–1171 were added at 1.5 or 2.0 μM concentrations to Xorbit depleted extracts upon entry of reactions into mitosis. For CLASP proteins CLASP1–1171 and CLASP1–662, only 0.4 μM was added due to dominant negative effects. As a negative control, an equivalent volume of CLASP elution buffer (100 mM HEPES, pH 7.5, 150 NaCl, 0.2% Tween-20, 1 mM DTT, EDTA, and 10 mM d-biotin (Sigma) was added to Xorbit depleted extract reactions.

### Spindle Imaging

Spindle reactions were spun onto coverslips, fixed and mounted for imaging, as previously described [Hannak and Heald,^2006^]. Images were collected with a fluorescence microscope (model BX51; Olympus, Center Valley, PA) with a dry 40 × NA (0.75) objective, a cooled CCD camera (model Orcall; Hamamatsu, Bridgewater, NJ) and Metamorph software (Molecular Devices).

### Centrosome Plus End Tracking Assays

2 μL of CSF extract was incubated with centrosomes purified from KE37 cells as previously described [Tournier et al.,^1991^], and incubated with various amounts of protein on coverslips. Exogenous purified EB1 was also added to a concentration of 4–5 μM. Coverslips were imaged using a Spinning Disk Confocal (Zeiss, Thornwood, New York). Similar experiments were also performed using metaphase-arrested egg extracts depleted of EB1 protein, using a similar immune-depletion method (using 25 μg of antibody) as for Xorbit described above.

## Results

### CLASP Is a Thin, Elongated Protein that Can Dimerize

**Fig. 1:**
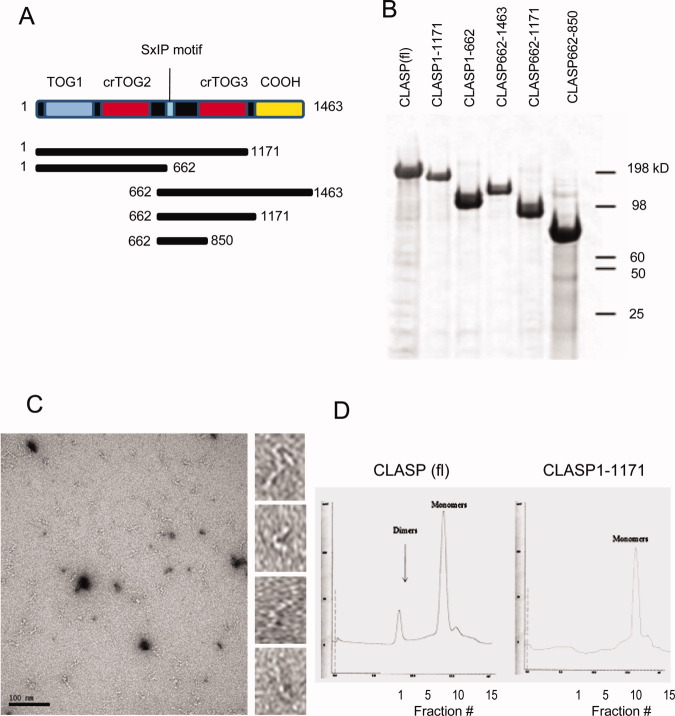
CLASP is a thin, elongated protein that can homodimerize. (**A**) Schematic of human CLASP1 domain architecture and the constructs generated for this study. (**B**) Representative sypro-ruby stained gel of purified full-length CLASP and smaller constructs, which were all expressed as StrepII-CLASP-GFP-10XHis fusions. (**C**) Electron micrograph of negatively stained, full-length CLASP showing elongated particles (see right panels for individual examples). (**D**) Elution profiles of two CLASP proteins after gel filtration. In the profile for full-length CLASP (left) there is a peak at fractions 12–18 corresponding to a MW of 180 kD that likely represents monomeric CLASP, and a second peak (arrow) for fractions 2–5 that corresponds to a protein MW of ∼350 kD and presumably represents dimeric CLASP. The elution profile for the CLASP1-1171 fragment (right) lacks this second peak, indicating that the C-terminal coiled-coil region is required for CLASP dimerization.

Full-length human CLASP1 protein and domain fragments were generated as C-terminal green fluorescence protein (GFP) fusions using baculovirus/insect cell expression. Using various informatics clues (Supporting Information Fig. S1), we designed a variety of constructs corresponding to different combinations of the known TOG domain (TOG1), two cryptic TOG domains (crTOG2 and crTOG3), SxIP, Ser-Arg-rich, and C-terminal coiled-coil regions in CLASP (Fig. 1A). Proteins were purified via a tandem affinity purification strategy using N-terminal StrepII and C-terminal 10× His tags (Fig. 1B). We first used electron microscopy of negatively stained CLASP samples to characterize the shape and size of the protein. Micrographs revealed CLASP as long and thin particles, about ∼10 nm in length. CLASP images suggested the presence of domains along the length of the molecule (Fig. 1C). However, the molecule appeared highly flexible, making image processing and the generation of class averages impossible. Despite this limitation, based on the apparent length of the CLASP particles, there seemed to be a mixture of monomers and dimers, although it was difficult to distinguish between actual dimerization and particles that were close together. The presence of a predicted coiled-coil region at the C-terminus of the protein (Supporting Information Fig. S1A), suggested that CLASP could indeed dimerize through this domain, similar to what has been shown for the *S. pombe* CLASP homolog [Al-Bassam et al.,^2010^].

To determine if CLASP can associate as a dimer through its C-terminal domain, full-length protein and CLASP1–1171 were subjected to gel filtration. CLASP1–1171 is identical to full-length CLASP except that it lacks the last 292 C-terminal residues (Fig. 1A). The gel filtration elution profile of full-length CLASP showed two major peaks, at fractions 2–5 and 13–16 (Fig. 1D). The earlier fractions of the elution profile correspond to a molecular weight of ∼350 kD, approximately twice that predicted for a monomer, suggesting that CLASP exists in an equilibrium between monomers and dimers. In contrast, CLASP1-1171 eluted in a single peak, a few fractions after the monomeric, full-length protein, reflecting a slightly smaller molecular weight. The fact that full-length CLASP formed dimers and CLASP1–1171 did not indicates that the C-terminus is responsible for dimerization. This result differs from experiments performed in vivo, in which CLASP2 was found to exist as monomers based on relative signal intensities of fluorescently tagged proteins [Emanuele et al.,^2005^; Drabek et al.,^2006^]. It is possible that CLASP can exist as either a monomer or dimer at equilibrium, which may be affected by its association with other binding partners at specific locations in the cell.

### CLASP Domains Contribute Differentially to MT Binding, Polymerization, and Bundling

**Fig. 2:**
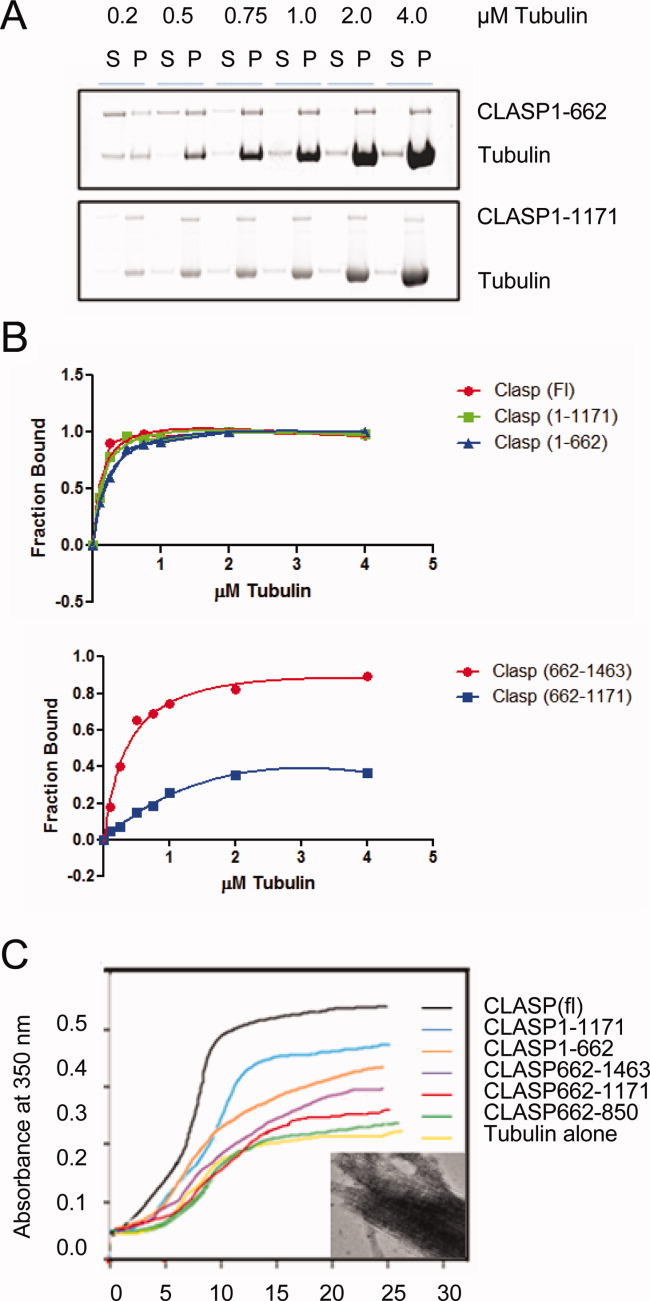
CLASP domains contribute differentially to MT binding, bundling, and rates of tubulin polymerization. (**A**) Representative gels of MT pelleting assays for CLASP1-662 and CLASP 1-1171 constructs. CLASP (0.5 μM) construct was added to 0–4.0 μM of taxol-stabilized MTs. Binding reaction mixtures were centrifuged, and both supernatant (S) and pellet (P) recovered and analyzed by SDS-PAGE (10% Bis-Tris) and visualized with sypro-ruby stain. (**B**) Affinity curves for the five different CLASP constructs derived from the pelleting assay gels. Band intensities were quantified using ImageJ (NIH) and plotted to construct an affinity curve from which the kd was estimated. Standard errors (not shown) were large and therefore only relative affinities should be evaluated. (**C**) 90° light scattering curves at 350 nm absorbance showing tubulin copolymerization with various CLASP constructs. Data was collected at 30 s intervals. Inset shows an EM micrograph of a MT bundle formed in the presence of the CLASP1-1171 construct, which also contributes to absorbance in this assay.

In order to characterize the MT affinities of CLASP and various fragments of the protein, binding curves were constructed using pelleting assays (Fig. 2A). A fixed concentration of protein (0.5 μM) was incubated with varying concentrations of taxol stabilized MTs (0.25–4.0 μM) (Fig. 2B). After a brief incubation, MTs were centrifuged and both the supernatant and pellet were recovered for analysis (Fig. 2B). Although absolute affinities were impossible to determine with much accuracy due to experiment variability, full-length CLASP, CLASP 1–1171, and CLASP 1–662 bound the MT lattice with relatively high affinity, with binding constants estimated to be in the range of 150–200 nM. CLASP1–662 contains the TOG1 and crTOG2 but lacks the second cryptic TOG domain (crTOG3), which may contribute to its apparent slightly weaker binding. CLASP662–1463 and CLASP662–1171 possessed less MT binding activity, as expected since there are fewer putative MT binding domains in those constructs. Interestingly, although the C-terminal coiled-coil domain (CLASP1171–1463) itself bound weakly to MTs (data not shown), CLASP proteins containing this domain bound more tightly to MTs. Altogether, these data suggest that all three TOG domains contribute to MT binding, which may be further enhanced by dimerization of CLASP though its C-terminal coiled coil.

The high binding affinity of CLASP proteins for the MT lattice predicted that CLASP would promote MT assembly. To test CLASP's effect on polymerization in the context of dynamic MTs, a 90° light scattering assay was used to measure bulk tubulin polymerization in the presence of different CLASP constructs (Fig. 2C). Proteins containing TOG1 and crTOG2 (CLASP (fl), CLASP1–1171, and CLASP1–662) all induced the same steep initial rate of tubulin polymerization, much faster than the rate of polymerization observed in the presence of other constructs lacking these domains or with tubulin alone. Differences in bundling activity (described below) of the various fragments likely also contributed to the observed differences in absorbances in this assay. Although the full-length protein generated greater bulk polymerization at steady state (plateau) than either CLASP1–1171 or CLASP1–662, the C-terminal domain missing from CLASP1–1171 had little, if any, effect on MT polymerization on its own, suggesting that the C-terminal domain is indirectly affecting MT polymerization, most likely by dimerizing CLASP via its coiled-coil region. In addition, the higher steady state polymerization observed in the presence of CLASP1–1171 compared to CLASP1–662 suggests that the second cryptic TOG domain (crTOG3) and/or the S/R-rich region present in the larger construct also promote MT polymerization. CLASP662–1463 stimulated MT polymerization more than CLASP662–1171, again consistent with the idea that the C-terminal dimerization domain generates an array of two crTOG3 domains, resulting in increased binding and polymerization activity. It is unclear if this domain's activity differs from that of the other TOG domains, but it likely contributes to both MT binding and polymerization (Figs. 2B and 2C).

In addition to the effect of CLASP proteins on overall MT polymerization, CLASP has also been seen to bundle MTs in vivo and it is thought that CLASP is essential for the formation of MT bundles required in a variety of cellular processes [Bratman and Chang,^2007^; Liu et al.,^2009^; Stramer et al.,^2010^]. In order to determine if CLASP also bundles MTs in vitro, samples of MT-CLASP mixtures from the polymerization reactions described above were prepared for EM analysis. The MT-CLASP samples were diluted 200-fold and adsorbed onto EM grids for staining and microscopy. Electron micrographs showed that CLASP constructs containing TOG1 and crTOG2 domains have potent bundling activity. Either CLASP1-1171 or CLASP1-662 resulted in massive MT bundles (Fig. 2C inset). In contrast, CLASP fragments lacking these domains were found to have dispersed MTs (Supporting Information Fig. S2A). Samples containing constructs CLASP662–1171 and CLASP662–1463 contained a few MT bundles, but the bundling activity was dramatically lower than that seen for TOG1 and crTOG2 containing proteins.

We reasoned that the bundling activity of CLASP might contribute directly to its stabilization of MTs, an idea that we tested using a cold MT pelleting assay. MTs were polymerized and bundled in the presence of CLASP1–662, and then placed on ice. Dynamic MTs depolymerize within a few minutes at this temperature, but certain stabilizing proteins can slow down or prevent depolymerization [Alushin et al., 2010]. SDS-PAGE analysis of MTs pelleted after cold treatment revealed stabilized MTs, bound to approximately stoichiometric amounts of this CLASP construct (Supporting Information Fig. S2B).

### CLASP SxIP Motifs Mediate MT Plus Tip Tracking Through EB1

**Fig. 3:**
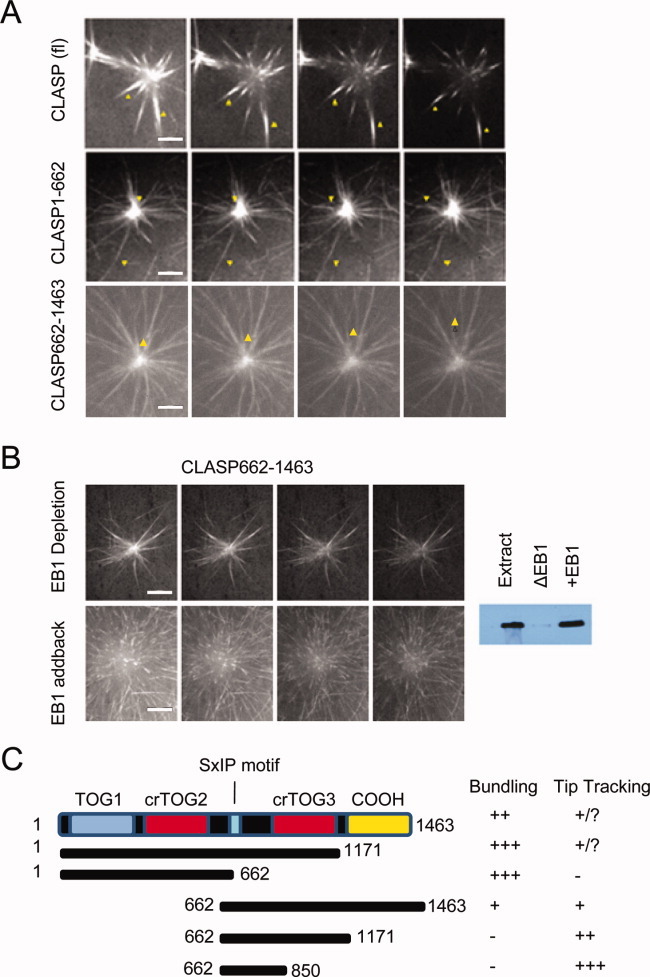
CLASP SxIP motifs mediate MT plus tip tracking though EB1. CLASP-GFP fusion proteins were added to human centrosomes in metaphase-arrested *Xenopus* egg extract. (**A**) Time-lapse images of full-length CLASP, CLASP1-662, and CLASP662-1463 in the assay. EB1 (4 μM) was added to enhance visualization of MT plus end association. Arrows indicate individual MTs or bundles (Supporting Information Movies S1–S3). (**B**) Centrosome tracking assay for construct CLASP662-1463 in EB1-depleted egg extract, and depleted extract to which recombinant EB1 was added back at the endogenous concentration of approximately 1 μM (Supporting Information Movies S4 and S5). (**C**) Summary of results for all tracking experiments with different CLASP constructs. All time-lapse images were collected at 2.5 s intervals. Scale bars = 10 μm.

Given that CLASP contains TOG domains similar to those present in the XMAP215 family that tracks MT plus ends autonomously, but also contains an SxIP motif that typically mediates EB1-dependent tip tracking of many other +TIPs, we used *Xenopus* egg extracts to investigate which CLASP domains are important for plus end tracking, and whether EB1 is required. GFP-tagged CLASP fragments were added to metaphase-arrested egg extracts, together with centrosomes that served as MT nucleation sites, and MT growth was imaged by time-lapse fluorescence microscopy (Fig. 3; Supporting Information Movies S1–S5). For full-length CLASP and the largest fragment, CLASP1–1171, plus end tracking was difficult to observe due to the bundling and stabilization of the astral MTs, but could be better visualized upon addition of up to 4.8 μM exogenous EB1 (Fig. 3A; Supporting Information Movie S1, and data not shown). CLASP1–662, which lacks the SxIP domain, did not track plus ends, despite the clear presence of dynamic MTs (Fig. 3A; Supporting Information Movie S2). Fragments containing the SxIP motif, including CLASP 662–1463 (Fig. 3A; Supporting Information Movie S3) tracked the growing ends of MTs, as did smaller domains containing the SxIP motif (CLASP662–1171 and CLASP662–805—Supporting Information Fig. S3 and Movies S4 and S5). However, the degree of tip tracking was variable, with the smallest fragment containing the SxIP domain (CLASP662–805) displaying the most robust activity, while the largest fragment (CLASP662–1463) tracked weakly. These results show that fragments containing TOG domains but lacking the SxIP domain bind to the MT lattice but fail to track plus ends, thus strongly indicating that CLASP's MT + TIP tracking ability is not mediated by its TOG domains, but rather by its SxIP motif, presumably through binding to EB1.

To further examine this dependency, we performed the assay in egg extracts immunodepleted of EB1. Under these conditions, CLASP662–1463, a protein fragment we observed to tip track, bound to the MT lattice but failed to tip track (Fig. 3B; Supporting Information Movie S6), an effect that could be rescued by adding back purified recombinant EB1 to endogenous levels of ∼1 μM [Kronja et al.,^2009^] (Fig. 3B; Supporting Information Movie S7). EB1 has been shown to enrich another TOG domain containing protein, XMAP215, on spindle MTs in egg extracts, suggesting that EB1 interaction and perhaps coordination with TOG domains and their respective activity is important for protein function. We observed that CLASP fragments containing TOG domains could still bind to the MT lattice in the absence of EB1 (data not shown), suggesting that while the TOG domains do not confer affinity for the plus end, they play an important role in MT binding (Fig. 3C).

### CLASP Interacts With Multiple Binding Partners Through Different Domains

**Fig. 4:**
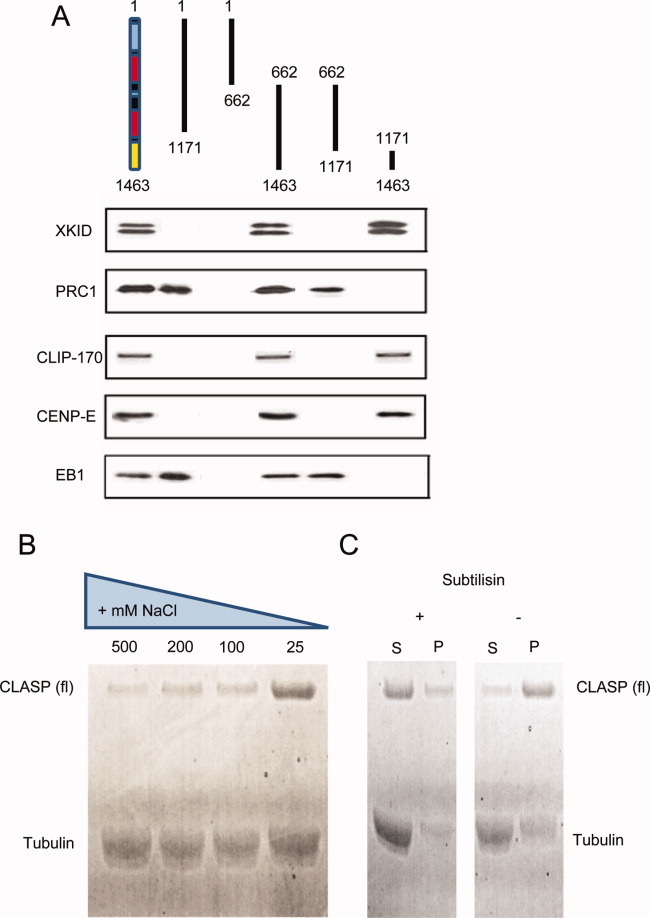
CLASP interacts with multiple binding partners through different domains. (**A**) Identification of CLASP interacting partners and binding domains. Full-length and shorter constructs of CLASP were used to pull down interacting proteins from *Xenopus* egg extract. For each CLASP construct, potential binding partners were separated by SDS-PAGE and probed by Western blot using available antibodies. Blots reveal the chromokinesin XKID as a novel partner that interacts with CLASP most likely through the C-terminal domain, similarly to XCENP-E and CLIP-170. PRC1 interacts with CLASP constructs that contain the S/R region and the putative crTOG3 domain. EB1 binds to all fragments containing the conserved SxIP motif. (**B**) Pelleting assays of full-length CLASP binding to MTs in the presence of increasing NaCl concentrations analyzed by SDS-PAGE (10% Bis-Tris) and stained with Sypro Ruby. (**C**) Pelleting assays of full-length CLASP binding to subtilisin treated (+) and untreated MTs (−) analyzed by SDS-PAGE (12% Bis-Tris) and stained with Sypro Ruby.

CLASP has been shown to associate with a number of binding partners, including CLIP-170 and CENP-E, through its C-terminal region [Hannak and Heald,^2006^]. To further define the CLASP region of interaction and identify other potential binding partners, we added our CLASP constructs to *Xenopus* egg extracts, retrieved them on beads using their StrepII tags, and then analyzed associated proteins by immunoblot (Fig. 4A). In addition to confirming already known partners, such as XCENP-E, CLIP-170, PRC1, and EB1, our assay also identified the chromokinesin XKID as a novel CLASP binding partner. Interestingly, while XCENP-E, CLIP-170, and XKID required the C-terminal coiled-coil domain of CLASP (residues 1171–1463) for interaction, EB1 and PRC1 interaction depended on the presence of the central region of CLASP that contains the SxIP motif and the crTOG3 domain. Whether the EB1 and PRC1 interaction domains are distinct or overlapping cannot yet be distinguished and will require generation of additional CLASP constructs.

### CLASP Interacts With MTS Primarily Through Electrostatic Interactions Involving the E-hook of Tubulin

To further probe the biochemical nature of the interaction between CLASP and MTs, we tested the hypothesis that the interaction is driven at least in part by electrostatic forces by assessing sensitivity to salt in MT pelleting assays. Addition of moderate amounts of salt (50 mM) resulted in significant loss of CLASP from the MT pellet, reflecting a decrease in affinity (Fig. 4B). To determine if this electrostatic interaction involved the acidic C-terminal tail (E-hook) of tubulin, MTs were treated with the protease subtilisin, which under controlled conditions cleaves off the C-terminal tail of tubulin. While full-length CLASP bound to native MTs, its affinity for subtilisin-treated MTs was greatly decreased (Fig. 4C), indicating the requirement of the tubulin E-hook for strong interaction with CLASP. However, a small amount of CLASP still pelleted with subtilisin-cleaved MTs, suggesting an additional interaction between this protein and the globular domain of tubulin within the MT lattice.

### CLASP Fragments Differentially Affect Spindle Assembly and Morphology in *Xenopus*

**Fig. 5:**
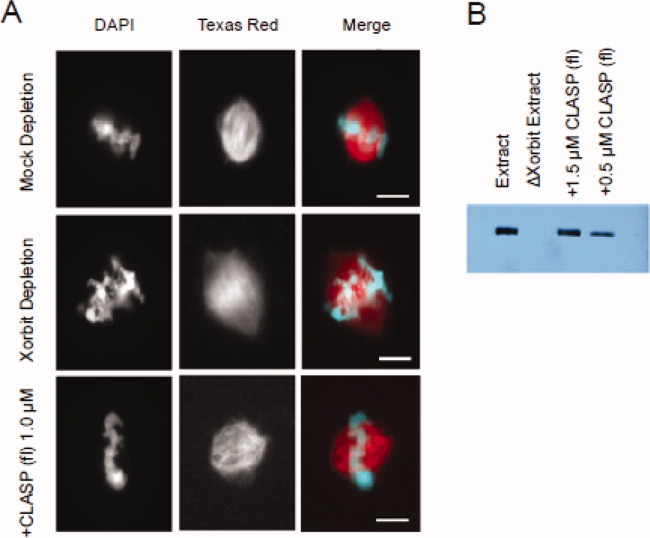
Rescue of Xorbit depletion with full-length CLASP. (**A**) Fluorescence images of spindles in egg extracts that were depleted with control antibodies (Mock depletion) or Xorbit antibodies followed by addition of buffer control (Xorbit depletion) or 1.0 μM full-length CLASP protein. (**B**) Western blot analysis of depletion and add-back reactions indicating that the endogenous CLASP concentration is approximately 1.5 μM.

**Fig. 6:**
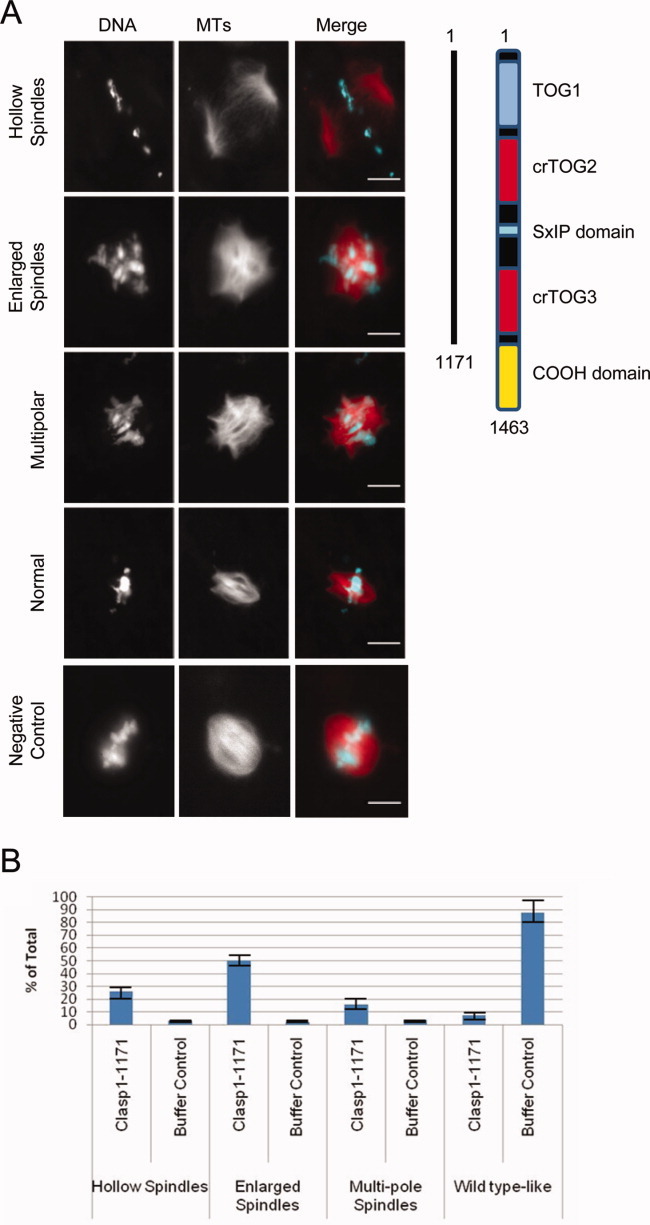
Exogenous CLASP protein lacking the C-terminal domain causes spindle MT polymerization and organization defects in *Xenopus* egg extract. (**A**) Representative images of major spindle phenotypes with addition of 0.2 μM CLASP1-1171 upon entry into mitosis. Scale bar = 10 μM. (**B**) Quantification of phenotype distribution comparing buffer control. Greater than 50 spindles were counted for each condition in three separate experiments.

**Fig. 7:**
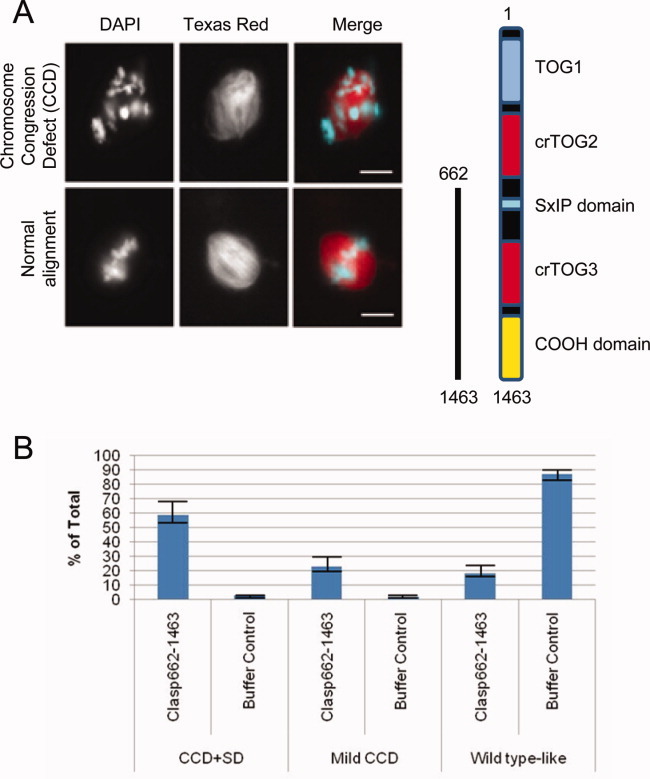
Exogenous CLASP protein lacking the N-terminal TOG domains causes defects in chromosome congression. (**A**) Representative images showing CCDs with addition of 0.2 μM CLASP662-1463 upon entry into mitosis. Scale bar = 10 μM. (**B**) Quantification of defects comparing buffer control. Greater than 50 spindles were counted for each condition in three separate experiments.

We used *Xenopus* egg extracts to assay the activity of various CLASP domains in the context of spindle assembly (Figs. 5–7). Of all the constructs tested, only full-length human CLASP, at the endogenous concentration of ∼1.0 μM, rescued spindle and chromosome congression defects (CCDs) in extracts depleted of the endogenous *Xenopus* homolog Xorbit (Fig. 5). This result illustrates, on one hand, the high degree of conservation between the human and *Xenopus* CLASP proteins and, on the other, the necessity for the full complement of CLASP domains for proper function in the spindle.

Interestingly, spindle morphology was altered upon addition of CLASP constructs at concentrations as low as 0.2 μM. The CLASP1–1171 construct (lacking the C-terminal domain) caused massive MT polymerization and bundling, enlarging spindles that often became round and multipolar (Figs. 6A and 6B). Similar but less dramatic effects were observed for the smaller construct CLASP1–662, which contains just the two N-terminal TOG domains (Supporting Information Fig. S4A). Another observed phenotype was “hollow spindles,” in which exogenous CLASP caused a shift in MT density from the spindle midzone to the poles (Figs. 6A and 6B). Because the C-terminal region is important for interaction with kinetochore and chromatin proteins including CLIP-170, CENP-E, and XKID, where it may localize CLASP to MT plus ends at chromosomes [Maiato et al.,^2005^; Hannak and Heald,^2006^], we believe these effects are due to mislocalization of CLASP activity, and thus, that the strong MT polymerizing activity of the N-terminal TOG domains must be localized correctly for proper spindle function.

CLASP662-1463, lacking TOG1 and TOG2, but containing the C-terminal domain, produced dominant negative spindle phenotypes reminiscent of but less dramatic than CCD previously observed upon addition of the C-terminal domain of CLASP (Figs. 7A and 7B) [Hannak and Heald,^2006^]. In addition to CCD phenotypes, mild spindle defects were observed. Altogether, these data highlight that all domains of CLASP contribute to its proper function, which requires its precise localization and the activity of multiple TOG domains in the spindle.

## Discussion

The data presented here obtained using recombinant human CLASP1 constructs provide insights into the function of CLASP domains and their physiological relevance to spindle assembly. Taken together, our biochemical data indicate that multiple domains contribute to MT binding and dynamics, including a crTOG3 domain in addition to the N-terminal TOG1, crTOG2, and S/R region. Studies with *S. pombe* CLASP have shown that the protein is able to bind both free tubulin and the lattice simultaneously, suggesting that CLASP can act as a tubulin shuttle, delivering free tubulin to the plus end of the MT [Al-Bassam et al.,^2010^]. However, it is not clear what domains within CLASP interact specifically with the MT lattice. A possible model is that the additional crTOG3, perhaps when arrayed in trans via dimerization through the C-terminal domain, creates another binding site that helps tether CLASP to the MT, while TOG1 and crTOG2 shuttle tubulin subunits to the growing end [Al-Bassam et al.,^2010^].

Our tracking assays show how in the presence of EB1, CLASP is enriched on the plus-ends of MTs, while without it, it is found all along the MT lattice. It is possible that CLASP activity is regulated by promoting or repressing these interactions, perhaps through post-translational modifications, as has been previously studied in filopodia formation [Wittmann and Waterman-Storer,^2005^; Watanabe et al.,^2009^]. In the context of the spindle assembly, CLASP might be enriched at the ends of dynamic MTs through interaction with EB1, thereby promoting kinetochore capture and the plus end polymerization of kinetochore fibers. The MT bundling activity may contribute to fiber formation, but this bundling activity must be tightly regulated, perhaps through modifications of the TOG domains. At the onset of anaphase, CLASP activity must be altered to promote the MT bundling observed in the central spindle.

Our in vitro studies demonstrate that CLASP is a potent MT polymerizing and bundling protein, while our results in *Xenopus* egg extracts show that these activities must be localized for proper spindle formation and maintenance. This is consistent with previous studies showing that introduction of the central region of CLASP1 has dominant negative effects on the spindle, leading to the formation of highly bundled, astral arrays of MTs that fail to attach to kinetochores [Maiato et al.,^2003^]. CLASP localization to kinetochores is thought to be mediated by interaction with the +TIP CLIP-170 and the kinetochore motor XCENP-E [Cheeseman et al., 2005; Galjart,^2005^; Tanenbaum et al., 2006] and our data indicate that chromokinesin XKID may promote CLASP localization and activity along chromosome arms. Additionally, a region flanking the TOG domains appears to bind PRC1, a known MT bundler and scaffold protein of the central spindle. CLASP can be viewed as bifunctional, acting both as a +TIP on dynamic MTs, as well as a potent stabilizer that induces MT bundles [Liu et al.,^2009^]. Localization of the protein likely directs activity for specific functions, and the dominant negative effects observed upon addition of CLASP domains to egg extracts illustrate the dramatic result of altering the localization and/or regulation of CLASP activities in the spindle. Unfortunately, because low levels of even the full-length CLASP had very strong effects on spindle morphology, we could not see bright enough GFP signals to meaningfully distinguish localizations of the different CLASP proteins or any changes occurring during spindle formation.

In summary, our study adds to the growing literature showing that CLASP is a potent and highly versatile regulator of the MT cytoskeleton involved in a number of different cellular functions. Key questions remain as to how CLASP operates as a MT polymerase, how domain activities are coordinated to promote MT binding, polymerization, and bundling, and how CLASP activities are regulated in the spindle to generate proper spindle MT architecture and dynamics.

## Supplementary material

Movie S1. GFP-tagged Full-length CLASP (1-1463) associates with MT plus ends but strongly bundles MTs when added to centrosomes incubated in metaphase-arrested Xenopus egg extracts.
