# Supplementary material for: Multiple Domains of Human CLASP Contribute to Microtubule Dynamics and Organization In Vitro and in Xenopus Egg Extracts
Source: Cytoskeleton (Hoboken). 2012 Jan 25;69(3):155–65. doi: 10.1002/cm.21005 (PMC3315288; doi:10.1002/cm.21005)
Supplement: Supplementary file 8 [file cm0069-0155-SD8.pdf]

SUPPLEMENTARY FIGURE LEGENDS

Figure S1

Design and expression testing of various CLASP fragments.

(A) Prediction of coiled-coil regions in CLASP1 using COILS (Lupas et al. 1991). Arrows indicate beginning and end of constructs in relationship to predicted coiled-coil regions (top). (B) Table of protein fragments generated and tested for expression/purification (bottom). (C, D) Sequence Alignments of TOG domains (C) and SxIP motifs (D) using (Simossis and Heringa 2005). The degree of sequence homology is indicated by color (blues – unconserved; green/yellow moderately conserved; reds – conserved).

Figure S2

MT binding and bundling for different CLASP constructs

(A) EM micrographs showing MT bundling in the presence of CLASP1-662, which is not nearly as prominent with CLASP662-1473 and CLASP662-1171 constructs. (B) Cold-pelleting assay with CLASP1-662. For experiments 2 (tubulin only control), half the amount of tubulin was used as compared to experiment 3, reflected in the different intensity of the tubulin bands. Samples were pelleted and analyzed via SDS-PAGE (10% Bis Tris) and stained with Sypro-Ruby).

Figure S3

MT ends tracking assays for CLASP constructs lacking TOG domains

Representative time-lapse images of CLASP662-1171 and CLASP662-805 that contain the SxIP motif but lack TOG1 and crTOG2 domains. Arrows indicate individual MTs or

bundles. Images were taken at 2.5 sec intervals. See movies S4 and S5 in supplemental material.

Figure S4

Dominant negative phenotypes caused by CLASP construct containing the N-terminal TOG domains

(A) Representative images of spindle phenotypes upon addition of 0.2 μM CLASP1-662 to spindle assembly reaction upon entry into mitosis. Scale bar = 10 μm. (B**)** Quantification of phenotype distribution comparing buffer control. Greater than 50 spindles were counted for each condition in 3 separate experiments.

SUPPLEMENTARY MOVIES

Movie S1. GFP-tagged Full-length CLASP (1-1463) associates with MT plus ends but strongly bundles MTs when added to centrosomes incubated in metaphase-arrested Xenopus egg extracts.

Movie S2. GFP-tagged CLASP1-662 fails to track MT plus ends in the assay, although it does bind MTs and promotes bundling.

Movie S3. GFP-tagged CLASP662-1463, containing the SxIP EB1 binding domain, but not N-terminal TOG domains, tracks MT plus ends in the assay.

Movie S4. GFP-tagged CLASP662-1171, containing the SxIP domain but not N or C terminal domains, tracks MTs plus ends, and binds weakly along centrosome MTs.

Movie S5. GFP-tagged CLASP662-805, containing a minimal SxIP EB1 binding domain is able to track MT plus ends in the assay.

Movie S6. GFP- tagged CLASP662-1463 does not track MT plus ends in EB1-depleted egg extract but does associate along the MT lattice

Movie S7. GFP-tagged CLASP662-1463 MT plus end tracking behavior is restored in EB1-depleted egg extract to which endogenous levels of EB1 were added back.

Figure S1

A

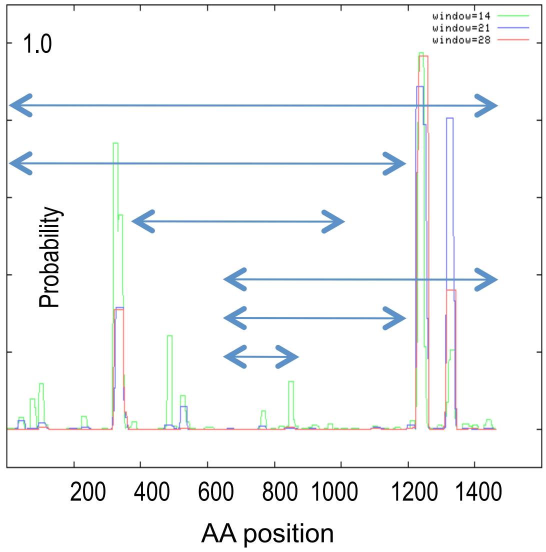

B

Construct	AA length	Expected Protein size	Predicted PI
CLASP1 (fl)	1463	168.52	10.21
CLASP 1-1171	1171	128.44	9.42
CLASP 1-662	662	72.77	9.22
CLASP 662-1463	801	88.50	8.36
CLASP 662-1171	509	55.70	9.15
CLASP 662-850	188	19.26	10.86

C

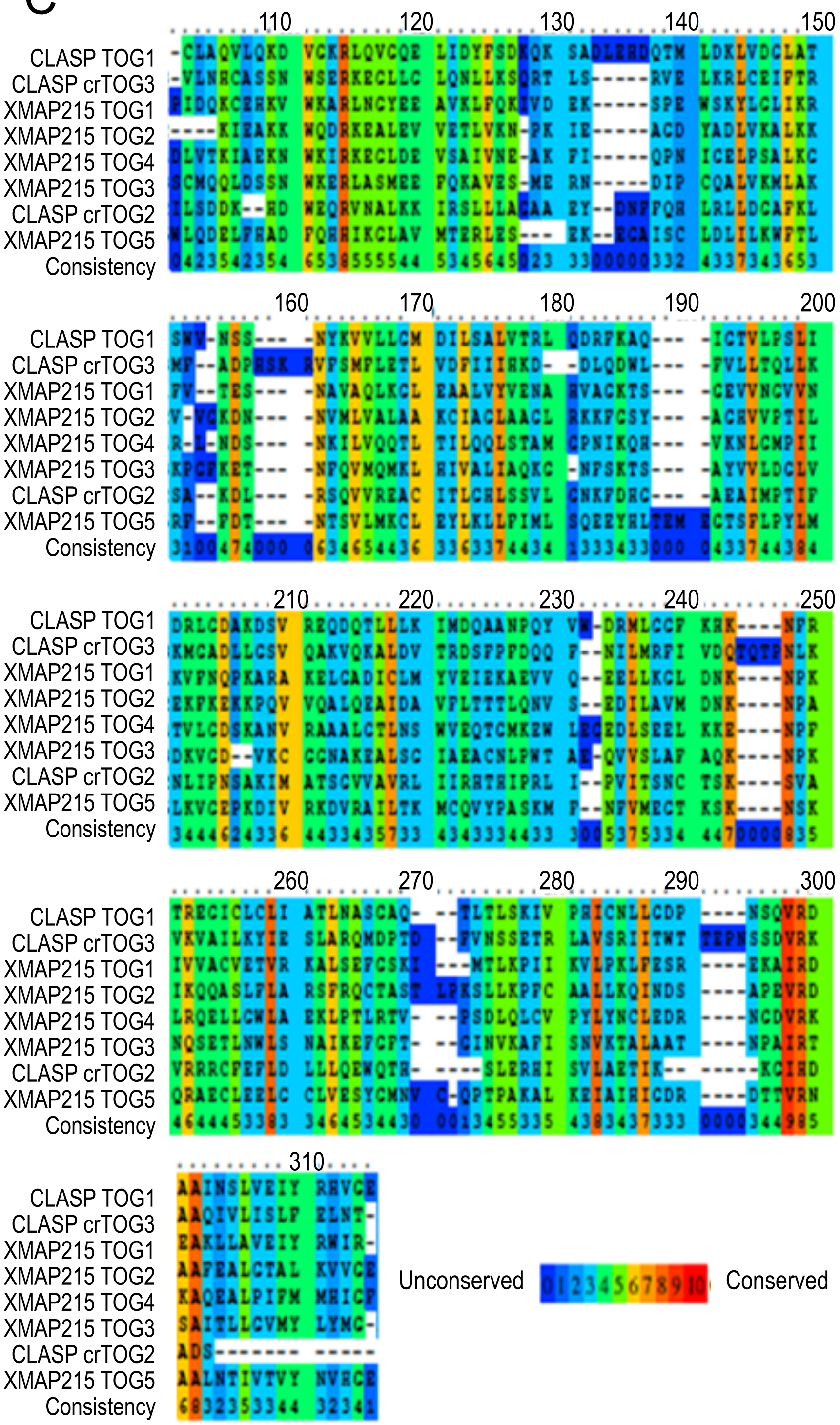

D

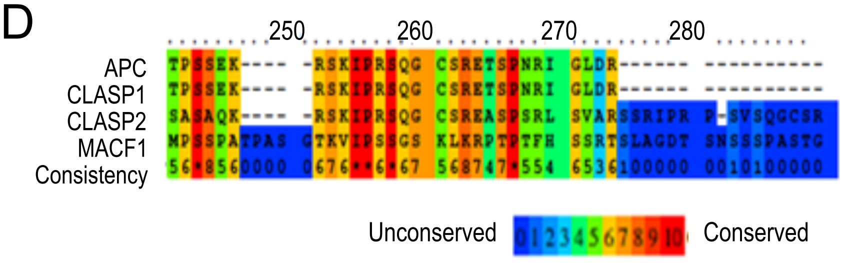

Figure S2

A

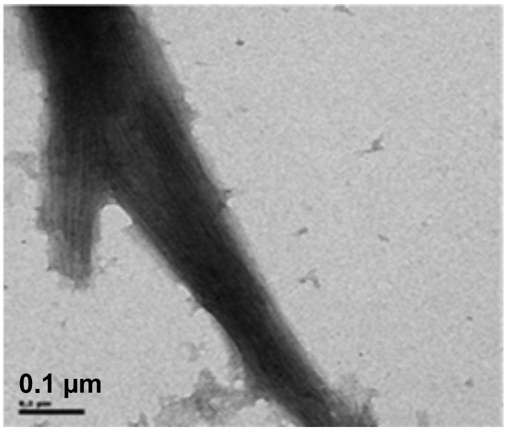

+CLASP1-662

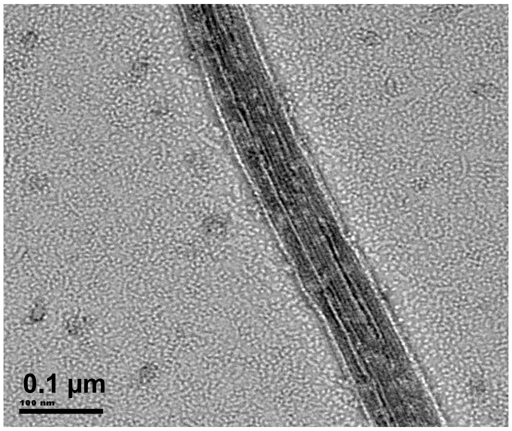

+CLASP662-1463

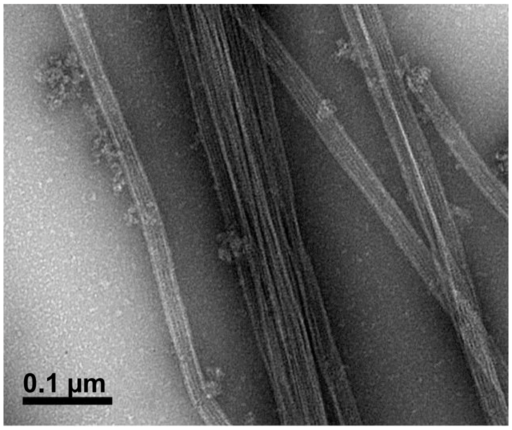

+CLASP662-1171

B

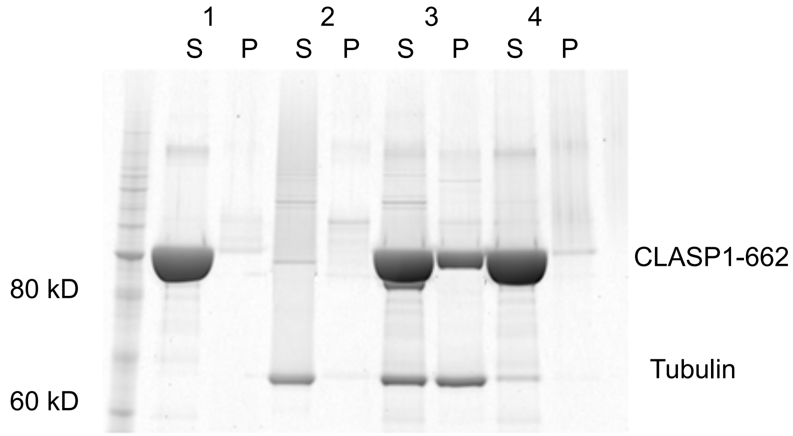

1: CLASP1-662 alone
2:Tubulin (polymerized at 37° C for 1 hour then placed on ice)
3:CLASP1-662 + Tubulin (polymerized at 37° C for 1 hour then placed on ice)
4:CLASP1-662 AT 37° C for 1 hour

Figure S3

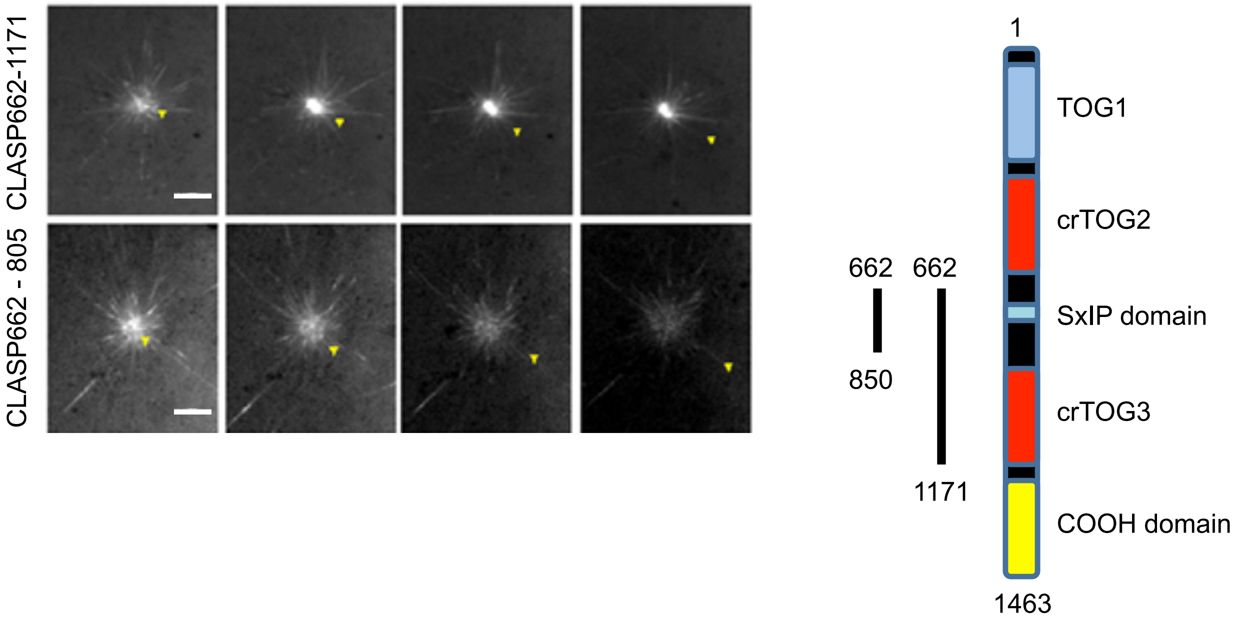
CLASP662-1171
CLASP662 - 805
662
850
662
1171
1
TOG1
crTOG2
SxIP domain
crTOG3
COOH domain
1463

Figure S4

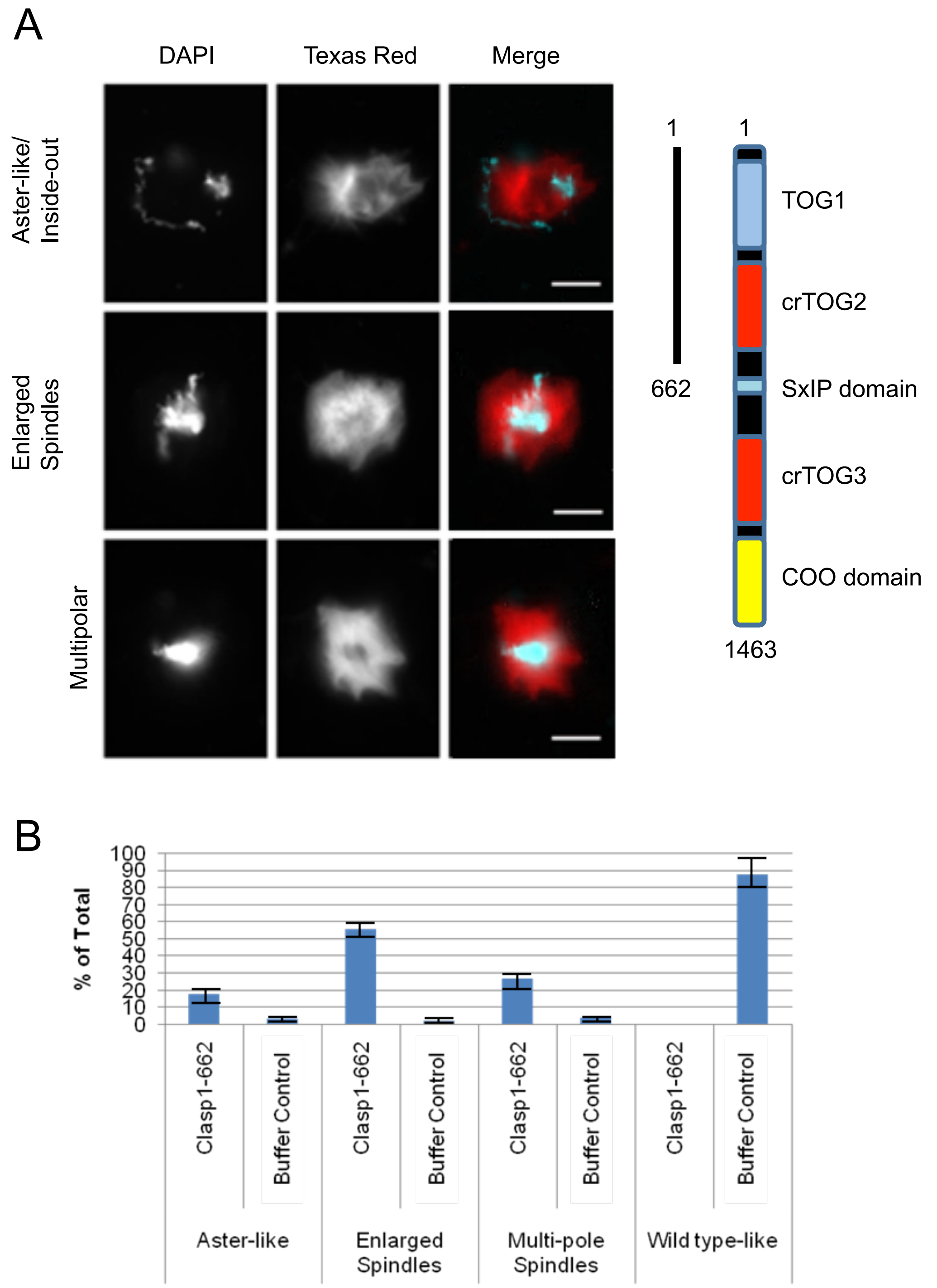
A
DAPI
Texas Red
Merge
Aster-like/ Inside-out
Enlarged Spindles
Multipolar
1
662
1
TOG1
crTOG2
SxIP domain
crTOG3
COO domain
1463
B
% of Total
100
90
80
70
60
50
40
30
20
10
0
Clasp1-662
Buffer Control
Aster-like
Enlarged Spindles
Multi-pole Spindles
Wild type-like
